# Charge transfer and ultrafast nuclear motions: the complex structural dynamics of an electronically excited triamine†
†Electronic supplementary information (ESI) available: Analysis of the spectrum (Fig. S1), the MOs of the ionic states (Fig. S2), and the structure of TMTAC and TMTAC^+^ conformers (Tables S1 and S2). See DOI: 10.1039/c5sc03042k


**DOI:** 10.1039/c5sc03042k

**Published:** 2015-10-19

**Authors:** Xinxin Cheng, Yan Gao, Fedor Rudakov, Peter M. Weber

**Affiliations:** a Department of Chemistry , Brown University , Providence , RI 02912 , USA . Email: peter_weber@brown.edu ; Fax: +1-401-8632594 ; Tel: +1-401-8633767; b Department of Chemistry , University of Missouri – Kansas City , Kansas City , MO 64110 , USA

## Abstract

Three ionization centers make 1,3,5-trimethyl-1,3,5-triazacyclohexane (TMTAC) an interesting model system to study intramolecular charge transfer (CT). Because the molecule assumes a *C*_s_ symmetric, axial–equatorial–equatorial (aee) conformation in the ground state, there are two distinct types of the nitrogen atoms. We discovered that either nitrogen atom can be ionized independently so that two molecular cations exist with different (localized) charge distributions in the Franck–Condon region. The initially localized charge can delocalize *via* CT, provided the molecule acquires a suitable structural geometry. These proper structures are all found to have a common structural motif that supports CT *via* through-space-interaction. The structural dynamics and the CT process in Rydberg-excited TMTAC, where the molecular ion core closely resembles the ion, were probed by time-resolved Rydberg fingerprint spectroscopy. When TMTAC is excited at 230 nm to the Franck–Condon region of the 3s Rydberg state, the two types of nitrogen atom Rydberg chromophores give rise to distinct binding energy peaks. The sequential molecular responses that follow the Rydberg excitation manifest themselves as time-dependent changes of the binding energy and are observed by ionization at 404 nm. A fast transition with 103 fs time constant was attributed to a motion that leads to a local minimum of the charge-localized state on the Rydberg potential energy surface. Because a large amount of energy is deposited into the vibrational manifolds, the molecule continues to sample the potential energy surface and eventually reaches a dynamic equilibrium between charge-localized and charge-delocalized states. The forward and backward time constants were determined to be 1.02 ps and 4.09 ps, respectively. The binding energy spectrum also reveals the existence of an equilibrium among several charge-delocalized states. Quantum chemical calculations were carried out to find the stable minima of the ground state and the ion state. The binding energies of the Franck–Condon structures and the relaxed ion structures were calculated using the Perdew–Zunger self-interaction corrected DFT (PZ-SIC) method to assign the spectra at time zero and at equilibrium, respectively.

## Abstract 2

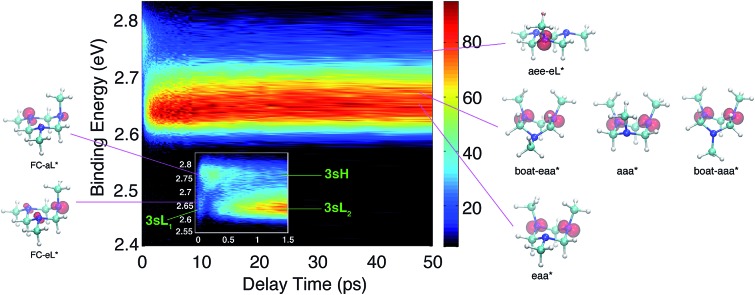
Time-resolved Rydberg fingerprint spectroscopy combined with quantum chemical calculations reveals the complex structural dynamics and charge transfer in real time.

## Introduction

Charge transfer (CT) processes are ubiquitous and important reactions because they are frequently found in biological systems including DNA and proteins,^1^–^3^ and are involved in light harvesting^4^ and functional devices.^5^–^7^ To study the time scales and the structural motions associated with CT, molecular model systems with several, possibly symmetry equivalent, amine groups are favorable. The present study builds on a series of reports on diamines^8^–^10^ and explores a triamine system where two of the nitrogen atoms are found to be symmetry equivalent, but distinct from the third. This opens up a fascinating interplay of charge transfer and structural motions involving chemically nearly identical functional groups.

**Fig. 1:**
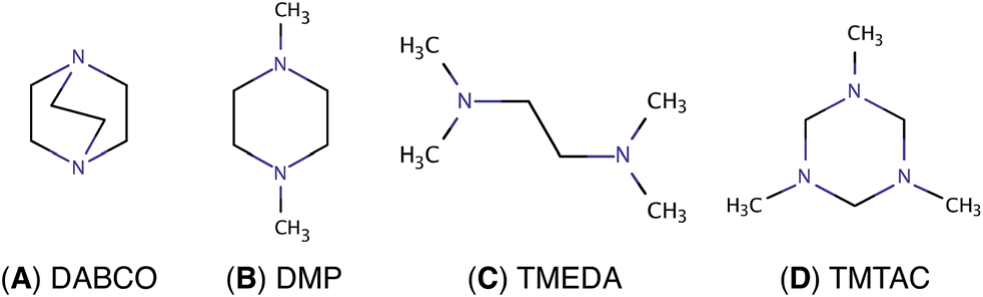
Chemical structures of (A) DABCO, (B) DMP, (C) TMEDA and (D) TMTAC.

In diamine molecules, variations in molecular structures as well as the length and number of carbon bridges give rise to intriguing CT dynamic phenomena.^8^–^12^ Depending on the arrangement of the nuclei, the nitrogen lone pair electrons may strongly or weakly interact in the ground state. Diazabicyclo[2.2.2]octane (DABCO, Fig. 1A) exemplifies a strong ground state interaction.^13^–^15^ Negligible interactions in the ground state are found in *N*,*N*′-dimethylpiperazine (DMP, Fig. 1B) and in *N*,*N*,*N*′,*N*′-tetramethylethylenediamine (TMEDA, Fig. 1C).^15^–^18^ These strong or weak interactions are manifest in the photoelectron and absorption spectra. Resonant Raman and transient absorption spectroscopic studies of the radical cations also indicate nuclear arrangements that lead to charge delocalization.^19^,^20^ Recently, we studied the ultrafast structural dynamics, *i.e.* the molecular skeleton motions, associated with the CT process when DMP and TMEDA are suddenly charged in a multiphoton excitation/ionization process.^8^–^10^ Using time-resolved Rydberg fingerprint spectroscopy (RFS),^21^–^23^ we find that the lone pair interactions, and with them the charge transfer, strongly depend on the conformational structures. We were able to monitor the structural evolutions of the molecular ion cores of DMP and TMEDA in real time after exciting an electron to a specific Rydberg orbital.

To extend the investigations of CT to the more complex triamines, we chose 1,3,5-trimethyl-1,3,5-triazacyclohexane (TMTAC, Fig. 1D), also sometimes referred as 1,3,5-trimethylhexahydro-1,3,5-triazine (TMHHTA), where three nitrogen atoms are embedded in a single, six-membered ring. Previous studies on TMTAC have focused on its conformational composition and stereodynamics in the ground electronic state.^24^–^27^ Although this ring molecule is structurally quite rigid compared to open-chain molecules, there are several conceivable combinations of the ring structure and the positions of the methyl groups. Recent gas phase electron diffraction^27^ and NMR studies^25^ suggest that the monoaxial chair conformer, *i.e.* the structure where the six-ring is in chair form and only one methyl group is in axial position, is the global minimum and the only substantially populated conformer in the ground electronic state.

In the present investigation we use time-resolved RFS in conjunction with quantum chemical methods to study the structural dynamics and CT when TMTAC is charged by optical excitation. Unlike DMP or TMEDA, where the nitrogen atoms are equivalent in the ground state, TMTAC has two different types of nitrogen atoms in the global minimum structure: one with the methyl group in axial position and two with methyl groups placed equatorially.^25^,^27^ Photoelectron spectra show two ionization potentials, with a splitting of 0.19 eV, that are attributed to ionization from the two different types of nitrogen lone pairs.^28^ It therefore seems possible to excite the molecule at different locations and to create Rydberg states that reflect different charge distributions in the molecular ion. Our aim is to investigate, with ultrafast time resolution and structural spectroscopy, the rich set of possible charge localization and delocalization phenomena that might arise in TMTAC upon excitation.

RSF is a special type of photoelectron spectroscopy whereby photoionization is achieved in a multi-photon process *via* Rydberg states. Because a Rydberg electron's binding energy (BE) is sensitive to the structure and charge distribution in the molecular ion core yet insensitive to the vibrational excitation, time-resolved RFS can probe the molecular structural dynamics unencumbered by vibrational motions.^21^–^23^ We have applied RFS to study the molecular structural dynamics in several systems, including the conformational equilibrium in hot flexible aliphatic molecules,^29^ the proton transfer reaction in large van der Waals clusters,^30^ the CT in a bi-functional molecule^31^ and in model molecules with equivalent chromophores.^8^–^10^ We have shown that changes in the BE can uncover transient structures in a CT reaction.^8^–^10^ This method has also been applied by other researchers to explore the relaxation pathways in conjugated systems such as azulene^32^,^33^ and 2-hydroxypyridine^34^ and to study the electronic structures of fullerenes.^35^,^36^ The molecular dynamics in a Rydberg state should closely resemble that of the cation in its ground state because the effect of the Rydberg electron on the chemical bonds of the ion core is very small. Since the ejection of a photoelectron is fast compared to the motions of the ion core, the photoelectron spectra reflect the molecular structure at the time of electron ejection. Structural motions can be followed by introducing a time delay between the optical excitation of the Rydberg state and the ionization. We discovered that the Rydberg electron binding energy is so specific to the electron charge distributions that it is possible to disentangle the complex dynamics in complicated molecular systems such as TMTAC.

## Experimental and computational details

The photoelectron spectroscopy apparatus has been described in detail previously.^9^,^37^,^38^ Ultrafast laser pulses were generated by a two-stage amplifier (a regenerative amplifier followed by a single pass amplifier, Coherent Legend Elite Duo). The two-stage amplifier produced 35 fs fundamental pulses at 808 nm with a 5 kHz repetition rate. 90% of this fundamental was used to generate 230 nm pulses by an optical parametric amplifier (OPA, Coherent Opera SOLO), while 10% was upconverted to the second harmonic (404 nm) using a Beta Barium Borate (BBO) crystal. The pulses at 230 nm were used as the pump to excite the molecules while the 404 nm pulses served as probe pulses to ionize them. The photoelectrons were detected with micro-channel plates (MCPs). The time-of-flights of the photoelectrons were recorded, analyzed and converted to kinetic energies to give the photoelectron spectra. The dynamical evolution of the excited molecule was monitored by introducing a variable delay time between the pump and probe pulses. Time zero, *i.e.* the time where the pump and probe pulses overlap in time, was determined by monitoring the two-color mass signal from the OPA pulse excitation and second harmonic ionization of the DMP molecule. The cross-correlation time was measured as 92 (3) fs at FWHM. The wavelengths were measured by a monochromator (SPEX Industries Inc., 270 M), which was calibrated using mercury atomic lines. The energies of the pump and probe laser pulses were 0.9 μJ and 6.0 μJ, respectively. The laser beams were focused onto the molecular beam with a 500 mm concave mirror. The peak intensities at the focus of the pump and probe pulses were estimated as 3.1 × 10^11^ W cm^–2^ and 8.2 × 10^12^ W cm^–2^, respectively. The intensities in the interaction region were determined by assuming Gaussian beam focal spot sizes. Imperfections cause the true focal spot sizes to be larger than calculated, so these results should be considered as the upper limits.

TMTAC was seeded in 1.1 bar of helium carrier gas and expanded through a 100 μm nozzle followed by a 150 μm skimmer. Anhydrous TMTAC (>97%) was purchased from Sigma-Aldrich and used without further purification. The TMTAC liquid was cooled in a temperature-controlled bath to 0 °C before entraining in the stream of helium, which reduces clustering in the molecular beam.

Conformeric minima in the neutral and cationic ground states were explored using Gaussian 09.^39^ Initial conformeric geometries of the TMTAC molecule were generated by creating a combination of the ring conformation (chair, boat and twist) and the positions of the methyl groups (equatorial and axial). Density functional theory (DFT) with the B3LYP functional^40^,^41^ and the PBE0 functional^42^,^43^ (also known as PBE1PBE in the Gaussian 09 program) and Møller–Plesset perturbation theory to second order (MP2) were used to optimize the structures. Coupled cluster calculations with single, double and non-iterative triple excitations (CCSD(T)) were carried out for single point energies with MP2 optimized structures. Unless specified otherwise, the Aug-cc-pVDZ basis set^44^,^45^ was used in all basis set based calculations. Frequency calculations were performed in the DFT and MP2 methods to ensure stable minima. The Rydberg-excited states and corresponding BEs were calculated using the Perdew–Zunger self-interaction corrected density functional theory (PZ-SIC),^46^,^47^ carried out with the GPAW program in real space with a uniform grid.^48^,^49^ The PZ-SIC method to calculate Rydberg states has been shown to give good results for several small to medium size monomers as well as large molecular clusters.^9^,^10^,^50^,^51^ The estimate of the Rydberg electron orbitals was obtained from a PZ-SIC calculation in the ground state at specific optimized molecular structure with the local density approximation (LDA) functional.^52^ The spin density, *i.e.*, the difference in density of electrons with spin-up and that with spin-down, was calculated with the corresponding orbitals. Previous work on the Rydberg states of big molecular clusters has shown the ability of the spin density to present the highly localized hole, corresponding to a positive charge, on one of the nitrogen atoms in the system after excitation of an electron to the Rydberg orbital.^51^ The energy of the highest singly occupied molecular orbital (SOMO) from calculating orbitals of the triplet ground state was used to estimate the BEs of the lowest energy excited state, in this case the 3s Rydberg state. In all the PZ-SIC calculations, the side length of the cubic simulation cell was set as 25 Å with a uniform grid size of 0.15 Å. Structures and molecular orbitals were visualized with GaussView 5.^53^ Contour plots of the spin densities were created with VMD.^54^

## Results and discussion

**Fig. 2:**
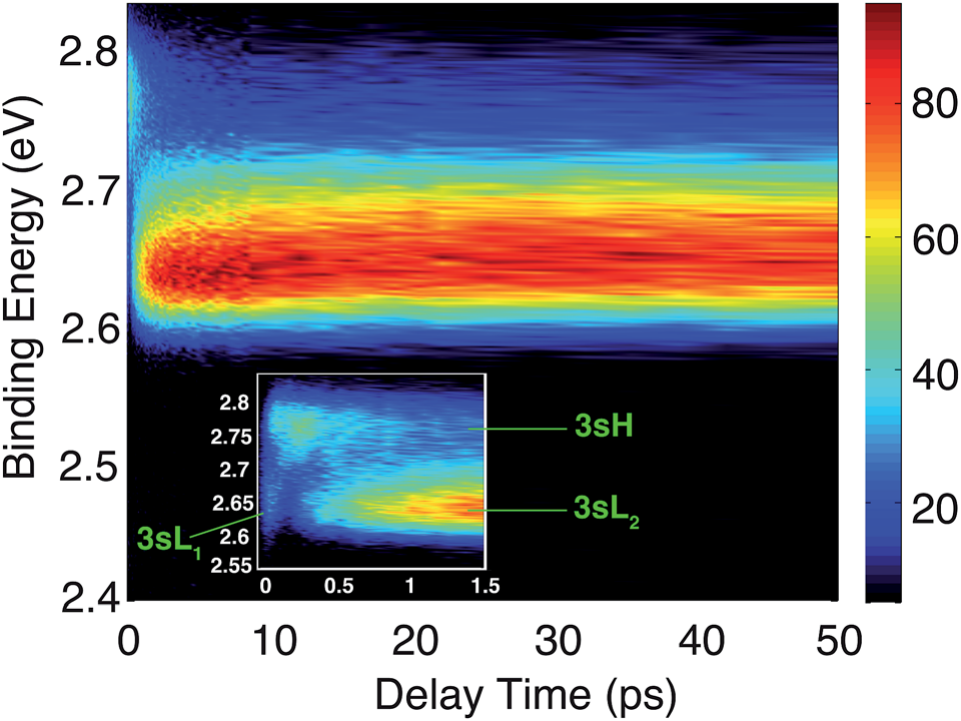
Contour plot of the time-dependent photoelectron spectrum of TMTAC, obtained by exciting at 209 nm and ionizing with 404 nm. The color bar shows the intensity in arbitrary units. The inset zooms in on the early delay time region. The BE is obtained by subtracting the measured photoelectron kinetic energy from the energy of the ionizing photon.

The time-resolved photoelectron spectrum is shown in Fig. 2. A detailed spectrum at early delay times is presented as an inset. As found in studies of other tertiary amines,^55^,^56^ the 3s Rydberg states are observed at BEs in the range of 2.55 to 2.85 eV. There are two component peaks, labeled 3sL and 3sH where L and H denote the lower and higher BE, respectively. The lower BE peak itself appears to be comprised of two components. One (3sL_1_) appears at time zero and has very short lifetime; the other (3sL_2_) has an initially slowly rising intensity, leading to a plateau after about 5 ps. These two peaks are likely from two different molecular structures that accidentally have similar BEs. The 3sH and 3sL_1_ peaks appear simultaneously at time zero, indicating that the pump laser pulse excites both states.

Two scenarios could account for the existence of the two peaks with early rise times: one is that two (or more) conformers are present in the molecular beam and excited at the same time; the other is that the pump laser excites non-equivalent nitrogen atoms of a single conformer. Based on the spectra alone, one cannot exclude either of the two explanations.

Since the BE of Rydberg state contains structural information of the molecular ion core, we fitted the spectra at each delay time point to determine the peak center positions of the partially overlapping peaks. Two Gaussians were used to fit the 3sH and 3sL_1_ peaks at delay times less than 0.5 ps. The fits gave 2.66 (0.01) eV and 2.77 (0.01) eV near time zero. We notice that after the initial excitation, the center of the 3sL_1_ peak shifts toward higher BE, indicating a time-dependent change of the molecular structure. For delay times longer than 0.5 ps, which includes the 3sH and 3sL_2_ peaks, three Gaussians were required to cover the observed spectroscopic features. A shoulder in the high-BE region of 3sL_2_ appears gradually, indicating that additional structures emerge and contribute to the higher BE part of the broad 3sL_2_ peak.^56^ The fits give 2.63 (0.01), 2.67 (0.01) eV and 2.77 (0.01) eV for the peak centers after reaching the equilibrium. Values in the parentheses are the 3*σ* error of the fits. Details are provided with Fig. S1 of the ESI.†

Calculations of the neutral and cationic ground states were carried out with the objective to assign the observed spectroscopic features. Assuming that the effect of the Rydberg electron on the structure of the molecular ion core is negligible, the structures of neutral and cationic ground states should resemble those of the Rydberg state ion cores at time zero and at equilibrium, respectively. Structures of the neutral ground states were optimized using MP2 and DFT with the PBE0 and B3LYP functionals. They all give similar structures.

**Fig. 3:**
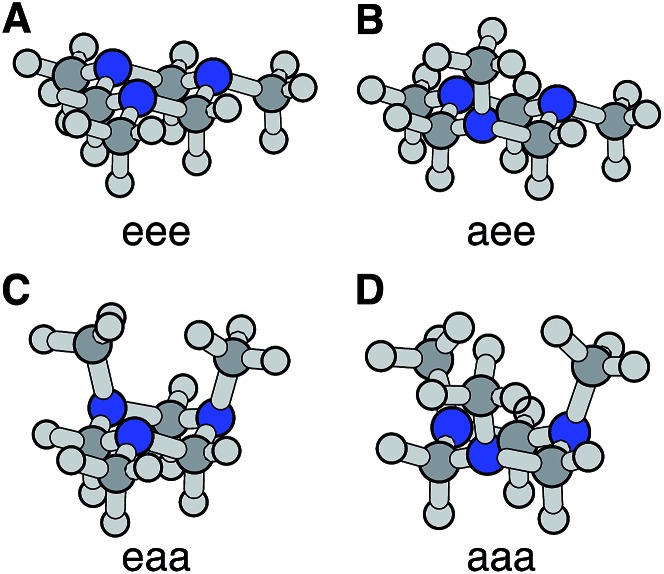
The four chair conformers of TMTAC in the ground electronic state. The labels e and a refer to the equatorial and axial positions of the methyl groups, respectively.

The optimized ground electronic state structures using MP2 are shown in Fig. 3A–D. The corresponding atomic coordinates are provided in Table S1.† The relative energies calculated at different levels of theory are listed in Table 1. The MP2 optimized structures were used in the single point energy calculations using the CCSD(T) method. According to the calculation, the global minimum in the ground state is the monoaxial aee conformer shown in Fig. 3B. The monoequatorial eaa conformer has the second lowest energy. All boat conformers become twisted after optimization and have very high relative energies (RE). Their structures and REs are therefore not shown here. These results are in good agreement with previous theoretical studies of the TMTAC conformers in the ground electronic state.^27^,^57^

**Table 1:** Calculated REs of TMTAC chair conformers shown in Fig. 3 at different levels of theory with the Aug-cc-pVDZ basis set. The energies are given in meV for the REs and in eV for the VIPs

Conformer	PBE0	B3LYP	MP2	CCSD(T)	VIP/eV
eee	115	109	153	148	8.43
aee	0	0	0	0	8.29
eaa	59	58	43	38	7.81
aaa	268	259	275	257	7.79

The vertical ionization potential (VIP) was calculated for each conformer using the MP2 optimized structures of the ground state at the CCSD(T)/Aug-cc-pVDZ level of theory. As listed in Table 1, the VIP of the global minimum in the ground state, the aee conformer, matches quite well with two independent experimental measurements of the VIP using photoelectron spectroscopy (8.33 eV measured in 1978 (ref. 58) and 8.46 eV measured in 1996 at room temperature^28^). The VIP of the second most stable conformer eaa, 7.81 eV, is below the experimental value. The computational results of the REs and VIPs suggest that the monoaxial aee structure of TMTAC is the only conformer present in the photoelectron spectroscopic experiments. This conclusion is supported by gas phase electron diffraction at 319 K (ref. 27) and NMR studies at 126 K,^25^ which also found only the monoaxial aee conformer. Therefore, it is reasonable to deduce that the monoaxial conformer is the only form that exists in the molecular beam where the molecules are generally well-cooled after the free jet expansion.

**Fig. 4:**
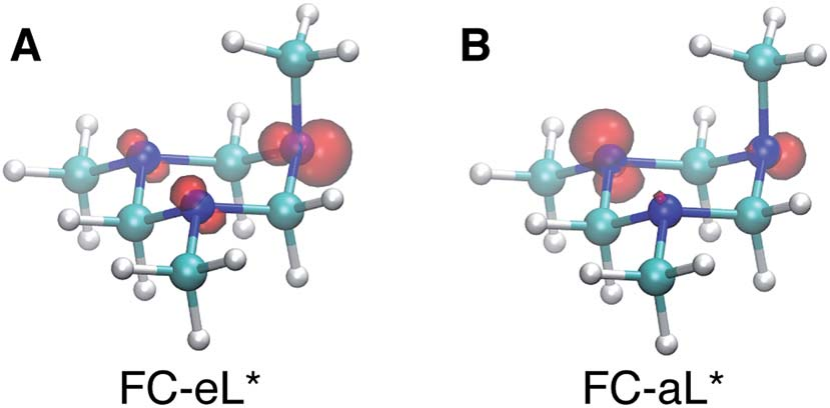
Plots of the spin density in the molecular ion core of Rydberg states with different charge distributions and the aee Franck–Condon (FC) structure. (A) FC-eL* shows the molecular ion core obtained by exciting an electron out of the equatorial lone pair (eL). (B) FC-aL* shows the molecular ion core reached by exciting an electron from the axial lone pair (aL). The rendered surface corresponds to a spin density of 0.2 Å^–3^, about 10% of the maximum value.

Based on the presence of only one conformer in the molecular beam, the peaks observed at early times, reflecting the Franck–Condon region of the transition from the ground state structure, are attributed to excitations of different types of nitrogen atoms. To gain a further understanding of the structural origins of the observed Rydberg peaks, we calculated the electron density distributions of the molecular ion cores of the Rydberg states reached by exciting an electron from either of the nitrogen atoms. To do this we simulated the excitations from the equatorial lone pairs or the axial lone pair separately using the PZ-SIC method. The spin density, which gives the difference in density of electrons with spin-up and spin-down, is shown as red contours in Fig. 4. It is quite apparent that excitation of an electron to the Rydberg orbital leaves behind a localized hole, corresponding to a positive charge, on different nitrogen atoms, depending on the lone pair that is excited. Fig. 4A and B show the effect on the molecular ion cores of excitation from the equatorial lone pair (eL) and axial lone pair (aL), respectively. A star denotes the 3s Rydberg excited states with ion core structures as given by the label before the star. A plus symbol denotes the ionic ground state or a molecular ion core structure that remains after one electron is elevated to the Rydberg orbital. For instance, FC-eL* refers to the 3s state with the Frank–Condon structure and the excitation is from the equatorial electron lone pair. FC-eL^+^ denotes the molecular ion core of the FC-eL* state. And as will be mentioned later, eaa* refers to the 3s state with the eaa^+^ ion core structure. Since we assume that the structures in the Rydberg states equal those of the ion states, eaa^+^ can refer to the ion or to the molecular ion core of the eaa* state.

To obtain the BEs of FC-eL* and FC-aL*, we calculated the optimized Rydberg orbitals of the triplet states. Because one of the electrons is already in the Rydberg orbital, using triplet states has been noted to give better results to calculate the BEs.^50^ Also, for the same reason, the energy of the SOMO can be used to estimate the BE of that Rydberg electron directly. This is very useful because the calculation of Rydberg states involving multiple centers is difficult to converge using the current implementation of the PZ-SIC method. Therefore, the orbital energy of the SOMO is used here to estimate the BE. The absolute energy of the Rydberg state is then calculated by subtracting the BE from the energy of the ion state obtained by the CCSD(T) single point energy calculation.

Using this approach, the BEs of FC-eL* and FC-aL* are estimated to be 2.51 eV and 2.62 eV, respectively. By comparing to the experimental values for 3sL_1_ and 3sH, respectively, we can assign the observed peaks at time zero to excitations from the equatorial lone pair and axial lone pair, respectively: the FC-eL* and FC-aL* states are observed in the RFS at BEs of 2.66 eV and 2.77 eV, respectively.

**Fig. 5:**
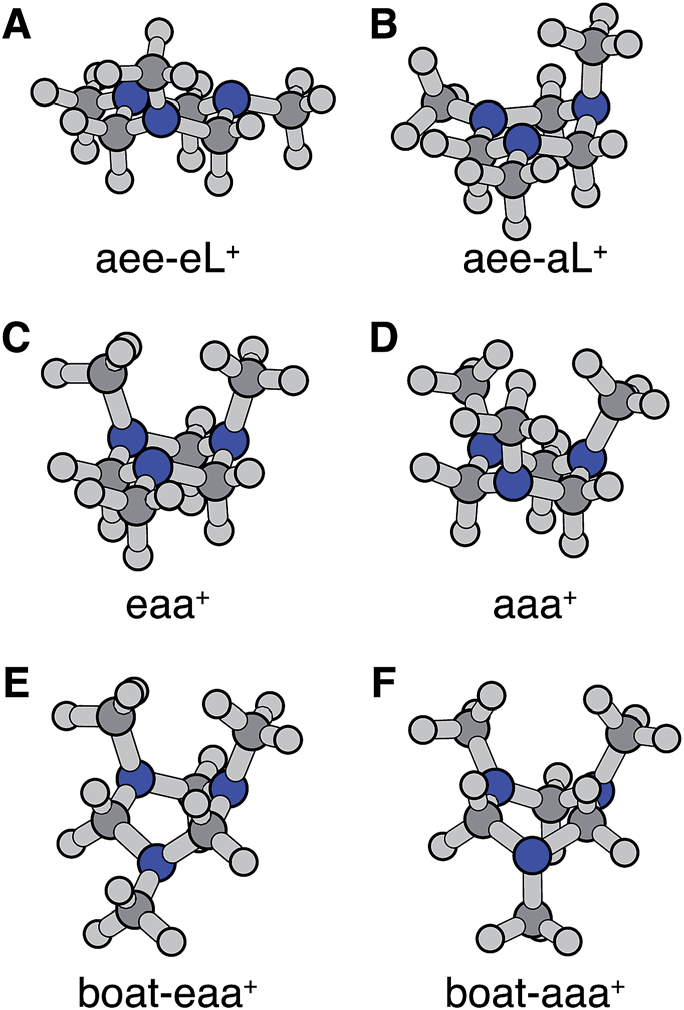
Stable TMTAC^+^ conformers in the ion ground electronic state. In (A) and (B) the aee-eL^+^ and aee-aL^+^ structures were obtained by optimizing the aee conformer with the nitrogen of the equatorial and axial lone pairs semi-planar as a starting structure on the ion potential surface, respectively. They are likely to be local minima to which the FC-eL^+^ and FC-aL^+^ configurations may converge on the ion potential surface. (C) is the global minimum in the ion state. (D)–(F) are the other three most stable ion structures.

Turning to the time-delay regions after the initial excitation, the molecular ion cores are expected to evolve and relax on the Rydberg potential surface. Because the Rydberg electron has a negligible effect on the structure of the molecular ion core, we use the ionic structures to estimate the corresponding cores of the Rydberg states. The six most stable TMTAC^+^ conformers were found by optimizing the structures on the ion potential surface starting with the stable chair and twisted boat structures of the neutral ground state. DFT and MP2 methods were used to optimize the ionic structures. For some structures, the two methods give different energy orders. In those cases, the CCSD(T) single point energies were calculated to decide which structure to use for the BE calculation. The optimized structures are shown in Fig. 5. The full coordinates of the optimized structures are given in Table S2 of the ESI,† together with some other high energy TMTAC^+^ conformers that are not further mentioned in the text. The eaa^+^ conformer (Fig. 5C) was found to be the global minimum in the ion ground state. The aee-eL^+^ structure (Fig. 5A) was obtained by optimizing the aee conformer with the eL nitrogen planar as a starting structure on the ion potential surface. With the aL nitrogen planar as starting point, the calculation converged to the aee-aL^+^ structure (Fig. 5B). From the Franck–Condon structure and excitation of either nitrogen atom, the molecule seems not to converge to the global minimum directly. The calculated relative energies are listed in Table 2.

**Table 2:** Calculated relative energies of the TMTAC^+^ conformers shown in Fig. 5 at different levels of theory with the Aug-cc-pVDZ basis set. The energies are given in the unit of meV

Conformer	PBE0	B3LYP	MP2	MP2-CCSD(T)
(A) aee-eL^+^	—^*a*^	—^*a*^	678	466
(B) aee-aL^+^	—^*a*^	—^*a*^	580	366
(C) eaa^+^	0	0	0	0
(D) aaa^+^	169	162	183	173
(E) boat-eaa^+^	47	49	125	121
(F) boat-aaa^+^	155	150	245	235

^*a*^The aee-eL^+^ and aee-aL^+^ structures are not stable at these levels of theory.

The BEs of the 3s Rydberg state were calculated for the most stable ion structures, assuming a negligible effect of the Rydberg electron on the structure of the molecular ion core. The total energies of the Rydberg states were calculated by subtracting the BE from the CCSD(T) single point energy of the ion states. The calculated BEs and relative energies are listed in Table 3. As was discussed earlier, the observed peak centers after reaching equilibrium were 2.63 (0.01) eV, 2.67 (0.01) eV and 2.77 (0.01) eV. Following the same trend as the calculated BEs of the FC structures, the calculated equilibrium structure BEs are systematically about 0.12 eV lower than the experimental measurements. After accounting for that, the calculated results are in excellent agreement with the experimentally measured values with the assignment as given in Table 3.

**Table 3:** Calculated BEs and relative energies (REs) of 3s Rydberg states associated with TMTAC^+^ structures shown in Fig. 5, and the experimentally measured BEs. The energies are given in the unit of eV

States	Cal. BE	Expt. BE	Cal. RE
aee-eL*	2.63	2.77	0.36
aee-aL*	2.74	—^*a*^	0.15
eaa*	2.52	2.63	0
aaa*	2.59	2.67	0.10
boat-eaa*	2.56		0.08
boat-aaa*	2.57		0.19

^*a*^The high BE peak was not observed in the spectrum.

From this comparison we conclude that while at time zero, in the Franck–Condon region, the FC-eL* and FC-aL* states are observed as 3sL_1_ and 3sH, respectively, the aee-eL* state with the corresponding aee-eL^+^ core structure (Fig. 5F) is responsible for the 3sH peak once the structure equlibrates. Noticing that the BE for the FC-eL* state is 2.66 eV and that it drifts higher after about 100 fs (Fig. S1†), the BE shift is attributed to a dynamic structural response to the optical excitation from the equatorial electron lone pair. This structural motion leads from the initially excited Franck–Condon structure of aee, FC-eL*, to the local minimum aee-eL* on the Rydberg potential energy surface. As described previously, the total 3s peak at equilibrium was fitted with three Gaussians. The major feature of the 3sL_2_ peak at equilibrium, with peak center of 2.63 eV, is now seen to arise from the global minimum, eaa^+^ structure (Fig. 5C). The boat-eaa^+^, aaa^+^ and boat-aaa^+^ structures may all contribute to the higher binding energy region of the 3sL_2_ peak, with a combined peak center of 2.67 eV. We notice that there also appears to be some dynamics within the broad peak 3sL_2_ since the middle peak in Fig. S1† appears only after 0.5 ps and the shape of the peak evolves with delay time. This likely reflects further structural dynamics between the eaa^+^, boat-eaa^+^, aaa^+^ and boat-aaa^+^ structures that we cannot fully resolve. We did not observe any spectroscopic feature with very high BE that might correspond to the aee-aL* state. Possible reasons for that will be discussed later.

**Fig. 6:**
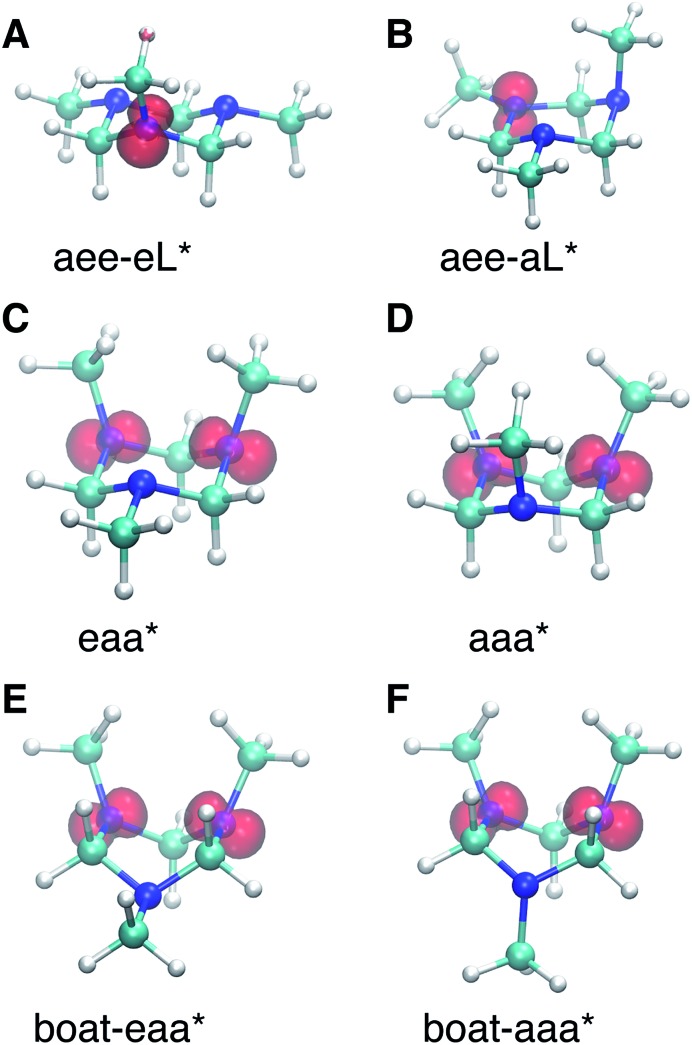
The spin densities of molecular ion cores of Rydberg states for equilibrium cationic structures. (A) and (B) Show the spin densities of the relaxed molecular ion cores reached by exciting the equatorial lone pair (eL) and the axial lone pair (aL) of the aee conformer, respectively. The charge is localized on the nitrogen atom where the electron is excited from. (C)–(F) are the spin densities with the most stable ion structures. The charge is delocalized between the two nitrogens with axial methyl groups. The rendered surface corresponds to a spin density of 0.2 Å^–3^ for (A) and (B) and 0.14 Å^–3^ for (C)–(F), about 10% of the maximum value.

The spin densities were calculated and visualized to reveal the charge distributions. For the aee-eL* and aee-aL* conformers, the spin densities suggest that the charge is localized on the nitrogen atom where the electron is excited (Fig. 6A and B). For all other cationic equilibrium structures the charge is delocalized between the two nitrogen atoms with axial methyl groups (Fig. 6C–F). The charge-delocalized structures, therefore, all share a structural motif of the two nitrogen atoms with axial methyl groups as shown in the figure. A molecular orbital analysis (Fig. S2†) shows that the electron lone pairs of the TMTAC^+^ conformers with two axial methyl groups are very close in space and may overlap effectively. As previously proposed,^14^ lone pair electrons in close spatial proximity can have a through-space-interaction (TSI) that stabilizes the cation. The conformers in Fig. 6C–F have such geometries that are suitable for TSI. The energy orderings of the SOMO and SOMO–1 of Fig. S2,† which show antisymmetric and symmetric combinations of the two lone pairs, respectively, are in agreement with the predicted TSI.^14^ This is in contrast to the cases of the DMP and TMEDA molecules, where the two nitrogen chromophores are separated by a distance of two carbon atoms and where the through-bond-interaction (TBI)^14^ dominates when the molecules assume a proper geometry.^8^–^10^

**Fig. 7:**
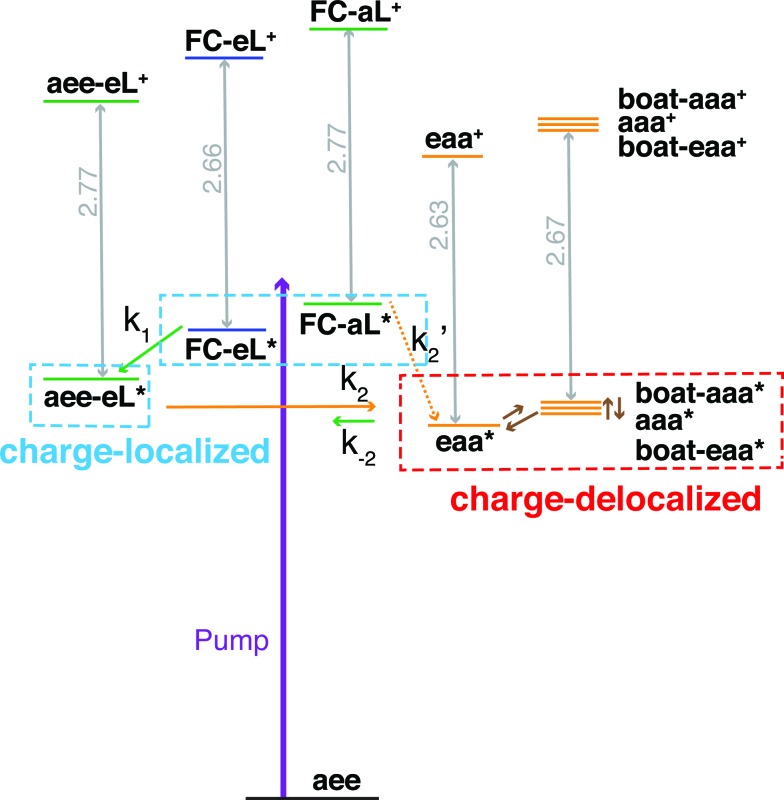
Schematic summary of the structural dynamics of TMTAC after excitation. Species marked in the blue and red boxes designate charge-localized and charge-delocalized states, respectively. The purple arrow illustrates the pump photon, which simultaneously excites the TMTAC molecule to the FC-eL* and FC-aL* states. Green and orange arrows represent transitions to the aee-eL* and eaa* states, respectively. The rate constants are shown above or under the arrows as *k*_1_, *k*_2_, *k*′_2_ and *k*_–2_. The corresponding time constants were obtained by fitting the time-dependent intensities to the kinetic model, see Fig. 8 and text for details. The orange dashed arrow labeled *k*′_2_ denotes a transition that is not separately observed as its spectral signature overlaps with the one labeled *k*_2_. Brown arrows without rate constants represent the dynamics between charge-delocalized states that was not spectrally separated.

Both TSI and TBI are quite sensitive toward the molecular structure. Optical excitation from the ground state does not likely access a geometry that is suitable for lone pair interactions. Once the system leaves the Franck–Condon region it can sample the multi-dimensional potential energy surface until it finds the most stable structure where the electron lone pairs have strong interactions and the charge can delocalize. Specifically, the equatorial lone pair (FC-eL*) state is found to initially evolve to the charge-localized aee-eL* state, resulting in a BE increase in the spectrum. On the other hand, the dynamics from the axial lone pair (FC-aL*) leads to a position where the lone pairs can interact and form the charge-delocalized structure belonging to eaa* (see scheme in Fig. 7). Because the BEs of FC-aL* and aee-eL* heavily overlap, this process is not separately observed. According to the calculation, there should also be a stable aee-aL* state, and it should have the highest BE among all the conformers. However, no such peak was observed in the spectrum. A possible reason is that the well of the aee-aL* state on the Rydberg potential surface is probably very shallow. Because the pump photon deposits quite a lot of energy into the vibrational manifolds, it might be very easy for the molecule to pass by this shallow well and directly access the deeper well of the eaa* state.

One should be aware that the transition from FC-eL* to aee-eL* involves structural dynamics rather than kinetics. However, because it is impractical to extract individual intensities for each time point along the dynamic process, we fitted the total time-dependent intensity to the simplified kinetics model so as to determine a time constant. For the subsequent processes, as the energy flows into other coordinates, the aee-eL^+^ structure evolves until a geometry is found where the lone pairs interact and the charge delocalizes. Because the aee-eL* state has a much higher relative energy (see Table 3), once these other, more stable states are found, there is a small probability for a return reaction. This is consistent with the spectroscopic observation, which finds that the 3sH peak intensity decays to less than 10% of its maximum value as the equilibrium is reached. The energy of the eaa* state can also flow into other coordinates. Because of the existence of more than one stable, delocalized structure, the 3sL_2_ peak is quite broad. Since the motion of the electron is fast, the picosecond time scale for reaching the equilibrium is attributed to the molecular structural rearrangements that are prerequisites for electron delocalization.

**Fig. 8:**
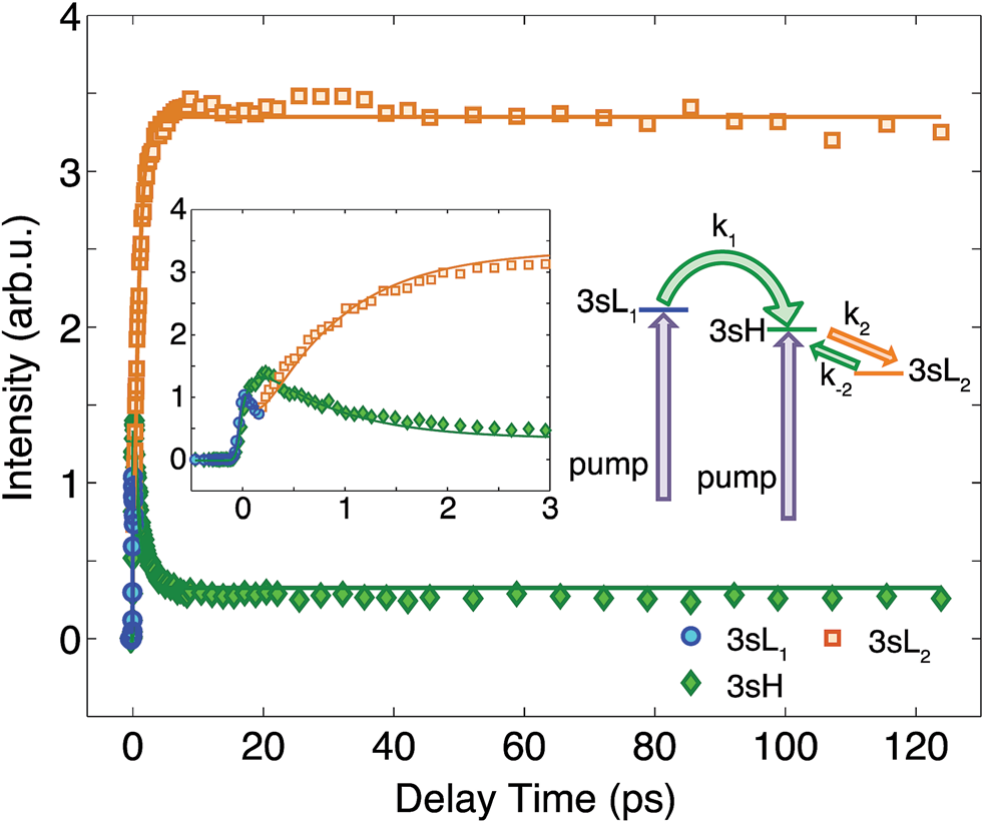
The time-dependent signals of the observed peaks. Symbols are the experimental data while solid lines represent the best fits using the Levenberg–Marquardt algorithm, assuming the simplified kinetic model shown as an inset. The boxed inset zooms in on the early delay time region. The kinetic model assumes first order kinetics for all processes. The purple arrows illustrate the simultaneous, direct excitation to the 3sL_1_ and 3sH states. Green and orange arrows represent kinetic transitions to 3sH and 3sL_2_, respectively. The colors of the symbols are matched to those in Fig. 7 and, for illustration purposes, the time-dependent 3sL_1_ and 3sL_2_ signals were given different colors for the different time ranges where they dominate even though they cannot be spectrally separated.

To determine the time constants for the dynamical processes uncovered in TMTAC we analyzed the time dependencies of the areas under each fitted curve of the individual component peaks. The so obtained time-dependent intensities of the 3sH and 3sL_1_ + 3sL_2_ peaks are shown in Fig. 8. Based on the analysis summarized in Fig. 7, we fitted the experimental data to the kinetic model shown as an inset in Fig. 8. The 3sL_1_ peak shows a very fast decay while the 3sH peak continues to rise after the excitation by the photon. Subsequently, the 3sH peak decays slowly while 3sL_2_ rises until reaching equilibrium. In the entire observation time window, after photoexcitation is complete, the intensity of the combined 3s signals remains constant. The fits yield rate constants *k*_1_, *k*_2_ and *k*_–2_ of 103 (39) fs, 1.02 (0.09) ps and 4.09 (0.72) ps, respectively. One should be aware that the kinetic model used to determine the time constants is a simplified version of the one shown in Fig. 7 as the various peaks partially overlap. The color scheme in Fig. 8 matches that of Fig. 7 to illustrate the correspondence of processes.

## Conclusions and perspectives

The ultrafast structural dynamics and CT in Rydberg-excited TMTAC were explored using time-resolved RFS in conjunction with computational simulations. Because there are two types of nitrogen atoms in the prevalent monoaxial aee conformer of the ground electronic state, and because the electron lone pairs have negligible interactions in the ground state, excitation with 230 nm pump pulses may place the molecule into either of two 3s states that have the charge localized on different nitrogen atoms: the ion cores that have, in their Franck–Condon region, the charges localized at nitrogen atoms with electron lone pairs in equatorial and in axial positions are observed at BEs of 2.66 eV and 2.77 eV, respectively. Once so prepared, the charge can delocalize in the molecule *via* CT provided the molecule assumes proper geometries. These proper structures are all found to have a common structural motif, namely two semi-planar nitrogen atoms, which supports TSI and charge delocalization. Interestingly, the charge only delocalizes between two nitrogen atoms; a delocalization amongst all three nitrogen atoms is not observed. This is likely because the TSI requires a direct interaction in space: the lone pairs have more sp^2^ hybridization and thus, apparently, are in better positions for the TSI with the charge delocalized between two nitrogen atoms instead of three.

The sequential molecular responses to the excitation were monitored by the time-dependence of the Rydberg electron binding energies. A fast transition with a 103 fs time constant was assigned to the molecular response to the excitation that leads to the local minimum aee-eL* on the Rydberg potential energy surface. The molecule keeps sampling the potential energy surface and eventually reaches the equilibrium between the localized states and delocalized states. The forward and backward time constants were determined to be 1.02 ps and 4.09 ps, respectively.

Once the structural dynamical processes are complete, the equilibrium structures show evidence for lone pair TSI and charge delocalization. Because the motions of electrons themselves are very fast, the pump–probe experiment does not directly probe the actual transfer of the charge. Instead, the BE spectra reveal, in real time, the responses of the ion core structures to the excitation and the structural dynamics associated with the CT from the charge-localized to the charge-delocalized structures. The ability of the PZ-SIC calculations with GPAW in real space with uniform grid to accurately calculate the BEs is extremely helpful in assigning the simulated structures to the observed spectra.

In this study of TMTAC, RFS shows its ability to probe the molecular structural dynamics with large amount of vibrational energy in the system, as is often needed to explore chemical reactions. Because the molecular ion cores of the Rydberg states closely resemble those of the ion and because the BE of a Rydberg state is sensitive to the structure and charge distribution of the core and insensitive to the vibrational excitation, RFS is a useful tool to study the structural dynamics of hot ions in real time. In TMTAC, we show that the two distinct BE peaks in the Franck–Condon region at time zero originate from different charge distributions with the same geometrical molecular structure. In previous studies on DMP, TMEDA and phenol derivatives we have found that the BE of Rydberg states is sensitive to a molecular ion core's charge distribution.^8^–^10^,^22^ But in those cases, the geometrical structures of the molecular ion cores were also different, so that it was impossible to separate the effect of the charge distribution from that of the nuclear geometry. To the best of our knowledge, the present study on TMTAC shows for the first time experimentally in a complex molecular system that the BE of Rydberg states is sensitive to the electron density distribution alone: even at time zero, where the molecular ion cores in the Franck–Condon region have the same structure, the binding energies of states with different charge distributions are distinct.

Future electron or X-ray diffraction experiments at similar molecular beam conditions would be beneficial to independently confirm the molecular structures and conformer distributions at equilibrium as well as the charge or electron distributions in the Franck–Condon region. It would be very interesting and important to see how those methods detect the configuration of the ion, including the nuclear arrangements and the electron distributions, compared to the RFS method.

## Supplementary Material

Supplementary information
