# Supplementary material for: Charge transfer and ultrafast nuclear motions: the complex structural dynamics of an electronically excited triamine
Source: Chem Sci. 2015 Oct 19;7(1):619–27. doi: 10.1039/c5sc03042k (PMC5952542; doi:10.1039/c5sc03042k)
Supplement: Supplementary file 1 [file SC-007-C5SC03042K-s001.pdf]

Electronic Supplementary Material (ESI) for Chemical Science.
This journal is © The Royal Society of Chemistry 2015

Charge transfer and ultrafast nuclear motions: the complex structural dynamics of an electronically excited triamine

Xinxin Cheng,[†ab] Yan Gao,[†a] Fedor Rudakov[c] and Peter M. Weber[*a]

[a] *Department of Chemistry, Brown University, Providence, RI 02912, USA*

[b] *Current Address: Max Planck Institute for the Structure and Dynamics of Matter, Atomically Resolved Dynamics Division, 22761 Hamburg, Germany.*

[c] *Department of Chemistry, University of Missouri - Kansas City, Kansas City, MO 64110, USA*

Supporting Information

This file includes:

- Analysis of the binding energy spectrum
  - Fig. S1
- Molecular Orbitals (MOs) of the ionic states
  - Fig. S2
- Structures of TMTAC and TMTAC$^+$ conformers
  - Tables S1 and S2

[*] Corresponding author. Tel.: +1-401-8633767; fax: +1-401-8632594.

E-mail address: peter_weber@brown.edu

[†] Equal contribution.

1. Analysis of the binding energy spectrum

To extract the peak center positions of the partially overlapping peaks, we fitted the 3s spectrum at each delay time point to Gaussians with flexible center positions and widths. For the time steps near time zero, two Gaussian functions sufficed to fit the overall intensities. For the time steps closer to the equilibrium, three Gaussian functions were necessary. For even longer delay times where equilibrium is reached, the intensity of 3sH becomes very small, so that fits with all parameters variable tend to ignore this peak. To extract meaningful information, we fitted the total intensity of those spectra with three Gaussians where the centers and peak widths were fixed. The fixed values were obtained from the averages of the fitted parameters at each delay time when the intensity of 3sH was still large enough to give an acceptable fit. The $3sL_2$ peak in Fig. 1 is broader than peaks we usually observe from a single conformer, indicating that more than one conformer is present and contributes to the signal. A selection of the spectra at specific time points, and the time dependences of the fitted peak centers and fitted peak widths are shown in Fig. S1. The peak center positions close to time zero and once equilibrium is reached were averaged, shown as lines in Fig. S1. The fits give 2.66 (0.01) eV and 2.77 (0.01) eV near time zero and 2.63 (0.01) eV, 2.67 (0.01) eV and 2.77 (0.01) eV after equilibrium is reached. Values in the parentheses are the 3σ error of the fits.

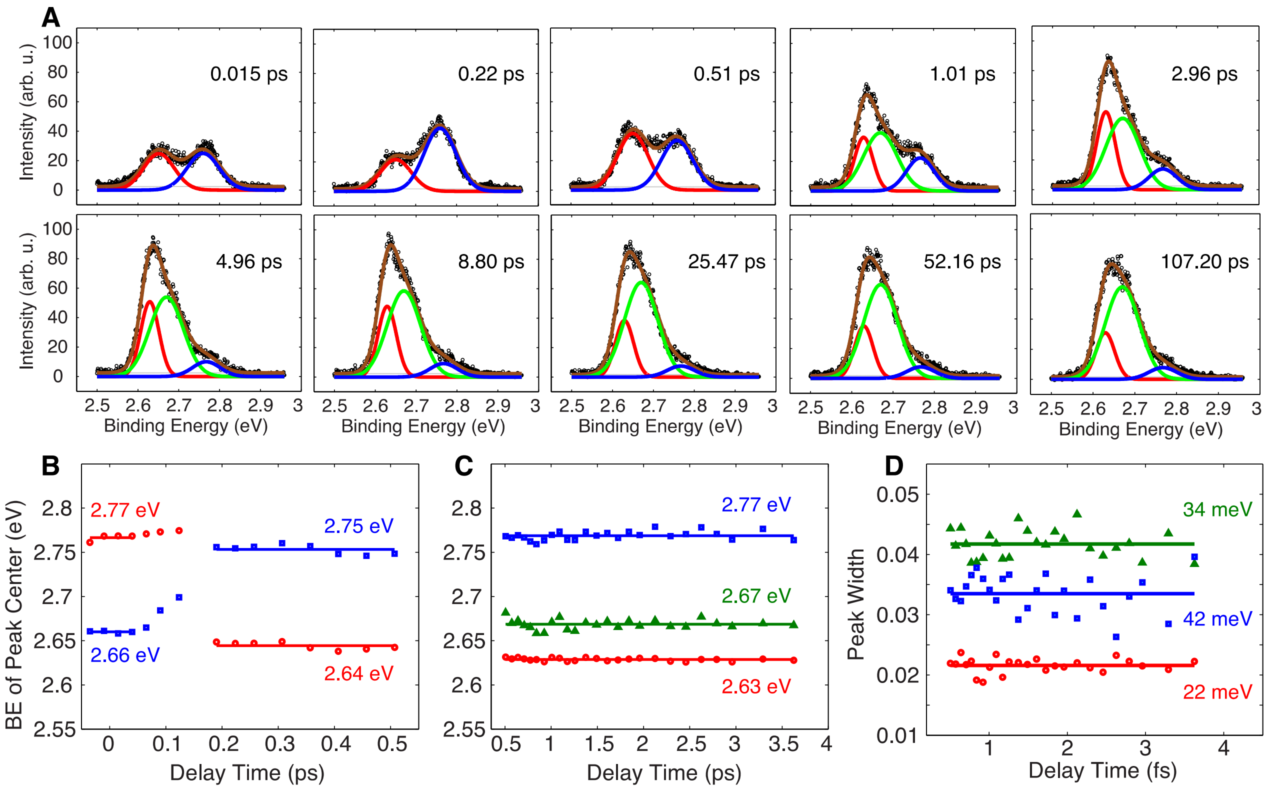

Fig. S1 The time-dependent signals. (**A**) The spectra and fitted curves at selected delay times. (**B**) BE peak center positions shown at short delay times. (**C**) BE peak center positions shown at long delay times when the equilibrium is reached. The spectra of only up to 4 ps were fitted for variable peak centers because after the equilibrium is reached, the intensity of 3sH becomes very small, so that fits with all parameters variable tend to ignore this peak. Therefore, those overall intensities of spectra after 4ps were fitted with fixed peak centers and bandwidths. (**D**) Peak widths for the three Gaussians. Symbols are the fitted peak center positions. Solid lines represent averages.

2. Molecular Orbitals (MOs) of the ionic states

The singly occupied molecular orbitals (SOMO) and SOMO-1 of eaa^+, aaa^+, boat-eaa^+ and boat-aaa^+ structures are shown in Fig. S2. All show the same antisymmetric and symmetric combination of the two lone pairs in the SOMO and SOMO-1, respectively, in agreement with the predicted consequence of the TSI.

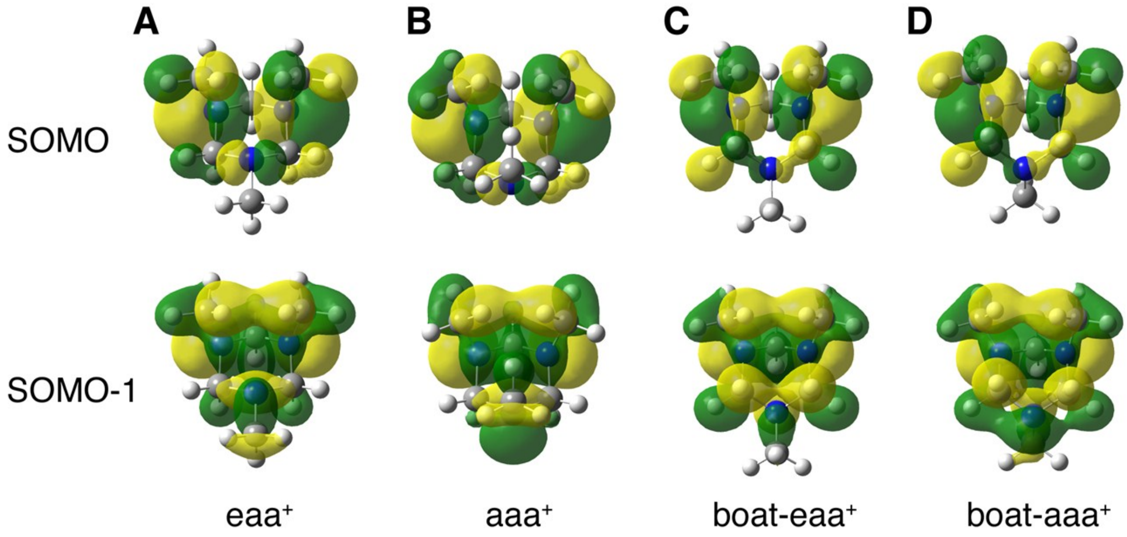

Fig. S2 SOMO and SOMO-1 of the TMTAC cations with the charge-delocalized structures.

3. Structures of TMTAC and TMTAC$^+$ conformers

Figs. 3 and 5 of the main text show different conformeric structures of TMTAC for the ground electronic state and for the molecular cation, respectively. Table S1 and S2 list the complete Cartesian coordinates for the conformers shown in the figures of the main text.

Table S1. Cartesian coordinates of the TMTAC conformers in the ground state.

Conformer eee

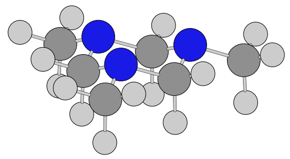

eee	Cartesian coordinates (Angstroms)		
Element	x	y	z
N	-1.197602	-0.691436	-0.394876
C	-1.186949	0.685285	0.088067
N	0.000000	1.382872	-0.394876
C	1.186949	0.685285	0.088067
N	1.197602	-0.691436	-0.394876
C	0.000000	-1.370571	0.088067
C	-2.388199	-1.378827	0.105050
C	0.000000	2.757655	0.105050
C	2.388199	-1.378827	0.105050
H	-1.217342	0.702833	1.217091
H	-2.084023	1.203211	-0.289251
H	0.000000	-2.406422	-0.289251
H	0.000000	-1.405666	1.217091
H	-3.287554	-0.867099	-0.269851
H	-2.433213	-1.404816	1.217294
H	-2.394706	-2.413556	-0.269851
H	2.084023	1.203211	-0.289251
H	1.217342	0.702833	1.217091
H	0.892847	3.280654	-0.269851
H	-0.892847	3.280654	-0.269851
H	0.000000	2.809632	1.217294
H	2.394706	-2.413556	-0.269851
H	3.287554	-0.867099	-0.269851
H	2.433213	-1.404816	1.217294

Conformer aee

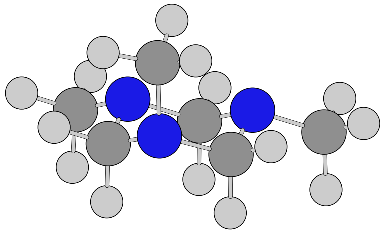

aee	Cartesian coordinates (Angstroms)		
Element	x	y	z
N	0.612121	1.445970	0.000000
C	-0.048249	0.912810	1.181384
N	-0.048249	-0.560511	1.200305
C	-0.734377	-1.033796	0.000000
N	-0.048249	-0.560511	-1.200305
C	-0.048249	0.912810	-1.181384
C	2.054659	1.154552	0.000000
C	-0.743638	-1.044523	2.391180
C	-0.743638	-1.044523	-2.391180
H	-1.094606	1.299715	1.189241
H	0.467911	1.273047	2.086041
H	0.467911	1.273047	-2.086041
H	-1.094606	1.299715	-1.189241
H	2.276130	0.073651	0.000000
H	2.504816	1.617947	0.891770
H	2.504816	1.617947	-0.891770
H	-0.739579	-2.136944	0.000000
H	-1.807238	-0.678728	0.000000
H	-0.738991	-2.145458	2.398795
H	-0.217782	-0.687637	3.290030
H	-1.800211	-0.695908	2.440007
H	-0.217782	-0.687637	-3.290030
H	-0.738991	-2.145458	-2.398795
H	-1.800211	-0.695908	-2.440007

Conformer eaa

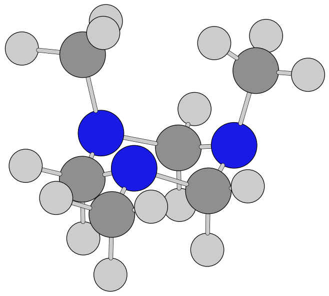

eaa	Cartesian coordinates (Angstroms)		
Element	x	y	z
N	0.903659	0.834273	0.000000
C	0.027866	0.810629	1.189085
N	-0.774994	-0.400243	1.253798
C	-1.528518	-0.532939	0.000000
N	-0.774994	-0.400243	-1.253798
C	0.027866	0.810629	-1.189085
C	1.707948	2.054404	0.000000
C	0.027866	-1.570712	1.631065
C	0.027866	-1.570712	-1.631065
H	-0.666607	1.683986	1.167386
H	0.656304	0.885905	2.092197
H	0.656304	0.885905	-2.092197
H	-0.666607	1.683986	-1.167386
H	2.351288	2.071922	0.893656
H	1.083514	2.976829	0.000000
H	2.351288	2.071922	-0.893656
H	-2.056073	-1.499230	0.000000
H	-2.276822	0.279822	0.000000
H	-0.582543	-2.478804	1.509775
H	0.957955	-1.680827	1.045860
H	0.297757	-1.483044	2.696105
H	0.297757	-1.483044	-2.696105
H	0.957955	-1.680827	-1.045860
H	-0.582543	-2.478804	-1.509775

Conformer aaa

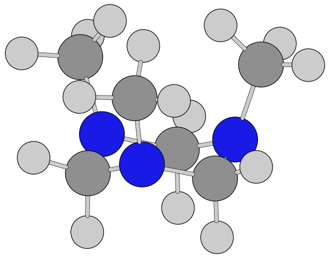

aaa	Cartesian coordinates (Angstroms)		
Element	x	y	z
N	0.000000	1.446174	-0.396401
C	-1.194191	0.689467	-0.792807
N	-1.252423	-0.723087	-0.396401
C	0.000000	-1.378933	-0.792807
N	1.252423	-0.723087	-0.396401
C	1.194191	0.689467	-0.792807
C	0.000000	1.960636	0.974456
C	-1.697960	-0.980318	0.974456
C	1.697960	-0.980318	0.974456
H	-1.219303	0.703965	-1.897745
H	-2.090850	1.207153	-0.414956
H	2.090850	1.207153	-0.414956
H	1.219303	0.703965	-1.897745
H	0.889941	2.596292	1.111344
H	0.000000	1.199196	1.774930
H	-0.889941	2.596292	1.111344
H	0.000000	-2.414306	-0.414956
H	0.000000	-1.407930	-1.897745
H	-1.803484	-2.068858	1.111344
H	-1.038534	-0.599598	1.774930
H	-2.693426	-0.527434	1.111344
H	2.693426	-0.527434	1.111344
H	1.038534	-0.599598	1.774930
H	1.803484	-2.068858	1.111344

Table S2. Cartesian coordinates of the $TMTAC^+$ conformers in the ion state.

Conformer aee-eL⁺

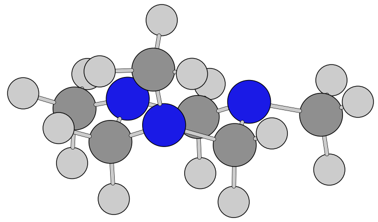

aee-eL⁺	Cartesian coordinates (Angstroms)		
Element	x	y	z
N	0.626108	1.273919	0.000000
C	-0.074756	0.877741	1.225085
N	-0.074756	-0.582540	1.196024
C	-0.785100	-1.046351	0.000000
N	-0.074756	-0.582540	-1.196024
C	-0.074756	0.877741	-1.225085
C	2.056293	1.475661	0.000000
C	-0.697971	-1.101160	2.429466
C	-0.697971	-1.101160	-2.429466
H	-1.088285	1.332606	1.180639
H	0.474978	1.244939	2.101982
H	0.474978	1.244939	-2.101982
H	-1.088285	1.332606	-1.180639
H	2.508565	0.462940	0.000000
H	2.355529	2.011731	0.909279
H	2.355529	2.011731	-0.909279
H	-0.775407	-2.145920	0.000000
H	-1.849039	-0.691480	0.000000
H	-0.650793	-2.198275	2.404024
H	-0.124389	-0.748064	3.298137
H	-1.754407	-0.784053	2.535014
H	-0.124389	-0.748064	-3.298137
H	-0.650793	-2.198275	-2.404024
H	-1.754407	-0.784053	-2.535014

Conformer aee-aL$^+$

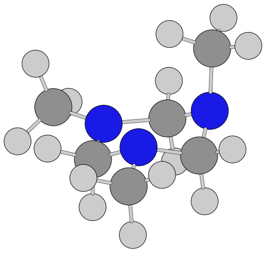

aee-aL$^+$	Cartesian coordinates (Angstroms)		
Element	x	y	z
N	0.023644	1.510247	0.428537
C	1.195386	0.744248	0.691757
N	1.188161	-0.597085	0.028845
C	-0.075726	-1.330923	0.008712
N	-1.182525	-0.447746	-0.278355
C	-1.167516	0.698740	0.647604
C	0.043582	2.161018	-0.896986
C	2.419064	-1.282916	-0.302190
C	-2.445364	-1.201319	-0.206438
H	1.275924	0.492855	1.770751
H	2.110485	1.262939	0.371254
H	-2.061476	1.307551	0.453450
H	-1.180174	0.370037	1.710518
H	0.043628	1.439259	-1.733543
H	0.931037	2.806894	-0.959644
H	-0.848892	2.795872	-0.980494
H	-0.004170	-2.135320	-0.740894
H	-0.124785	-1.806173	1.026760
H	2.559067	-1.203197	-1.397142
H	3.264145	-0.805426	0.210301
H	2.336648	-2.346550	-0.034178
H	-3.268831	-0.529195	-0.483152
H	-2.417296	-2.030658	-0.928589
H	-2.636833	-1.609892	0.806662

Conformer eaa$^+$

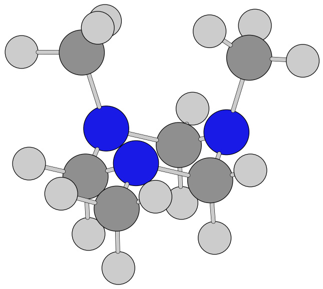

eaa$^+$	Cartesian coordinates (Angstroms)		
Element	x	y	z
N	0.337619	1.231352	0.000000
C	-0.391294	0.729141	1.178749
N	-0.391294	-0.728893	1.137069
C	-1.130807	-1.262879	0.000000
N	-0.391294	-0.728893	-1.137069
C	-0.391294	0.729141	-1.178749
C	0.408325	2.703133	0.000000
C	0.828406	-1.395222	1.582682
C	0.828406	-1.395222	-1.582682
H	-1.448980	1.070975	1.192724
H	0.122962	1.061670	2.092408
H	0.122962	1.061670	-2.092408
H	-1.448980	1.070975	-1.192724
H	0.961586	3.031689	0.891189
H	-0.593840	3.176370	0.000000
H	0.961586	3.031689	-0.891189
H	-1.123432	-2.359847	0.000000
H	-2.154924	-0.863302	0.000000
H	0.740553	-2.477121	1.418451
H	1.715106	-0.995718	1.056375
H	0.947042	-1.199856	2.659443
H	0.947042	-1.199856	-2.659443
H	1.715106	-0.995718	-1.056375
H	0.740553	-2.477121	-1.418451

Conformer aaa$^+$

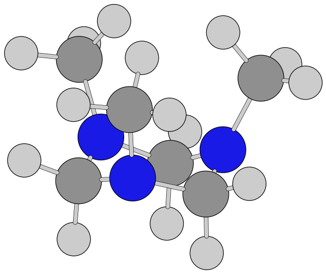

aaa$^+$	Cartesian coordinates (Angstroms)		
Element	x	y	z
N	-1.322985	-0.837492	0.000000
C	-0.470030	-0.965183	1.177432
N	0.739146	-0.112997	1.129949
C	1.600232	-0.431753	0.000000
N	0.739146	-0.112997	-1.129949
C	-0.470030	-0.965183	-1.177432
C	-2.184891	0.356550	0.000000
C	0.739146	1.233810	1.682438
C	0.739146	1.233810	-1.682438
H	-0.108907	-2.006867	1.230989
H	-1.036797	-0.716720	2.085099
H	-1.036797	-0.716720	-2.085099
H	-0.108907	-2.006867	-1.230989
H	-2.831176	0.321003	-0.888547
H	-1.636990	1.316835	0.000000
H	-2.831176	0.321003	0.888547
H	2.509622	0.184424	0.000000
H	1.842295	-1.505172	0.000000
H	1.776049	1.552574	1.862711
H	0.251152	1.966892	1.010281
H	0.187920	1.221123	2.633187
H	0.187920	1.221123	-2.633187
H	0.251152	1.966892	-1.010281
H	1.776049	1.552574	-1.862711

Conformer boat-eaa$^+$

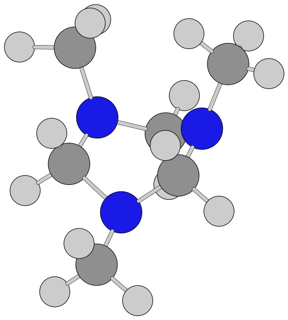

boat-eaa$^+$	Cartesian coordinates (Angstroms)		
Element	x	y	z
N	-0.082416	1.447135	0.000000
C	0.306363	0.686361	1.175570
N	-0.400580	-0.602409	1.125523
C	-1.315575	-0.746978	0.000000
N	-0.400580	-0.602409	-1.125523
C	0.306363	0.686361	-1.175570
C	0.487909	2.804025	0.000000
C	0.306363	-1.762896	1.648330
C	0.306363	-1.762896	-1.648330
H	0.011447	1.239315	2.084734
H	1.402407	0.478113	1.222527
H	1.402407	0.478113	-1.222527
H	0.011447	1.239315	-2.084734
H	1.595585	2.795795	0.000000
H	0.131362	3.338133	0.891548
H	0.131362	3.338133	-0.891548
H	-1.765537	-1.747263	0.000000
H	-2.071628	0.043630	0.000000
H	-0.359477	-2.635654	1.644945
H	1.220890	-1.982527	1.061762
H	0.613310	-1.543502	2.682506
H	1.220890	-1.982527	-1.061762
H	-0.359477	-2.635654	-1.644945
H	0.613310	-1.543502	-2.682506

Conformer boat-aaa$^+$

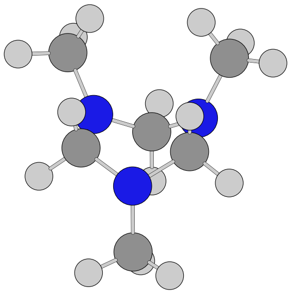

boat-aaa$^+$	Cartesian coordinates (Angstroms)		
Element	x	y	z
N	-1.467511	0.009715	-0.516092
C	-0.733186	1.250324	-0.513506
N	0.496330	1.156079	0.328316
C	0.519839	0.001474	1.211157
N	0.531967	-1.111616	0.271968
C	-0.572600	-1.090562	-0.731880
C	-2.570833	-0.167527	0.439450
C	1.740405	1.649426	-0.246863
C	1.801216	-1.665478	-0.174710
H	-1.368483	2.066643	-0.132313
H	-0.393978	1.501065	-1.535744
H	-0.103385	-1.007177	-1.733286
H	-1.119302	-2.046058	-0.675947
H	-3.144612	-1.055409	0.139150
H	-3.235332	0.704452	0.366607
H	-2.265089	-0.290814	1.495282
H	1.433923	-0.004467	1.818868
H	-0.365204	-0.017151	1.855999
H	2.550013	1.570791	0.490489
H	2.009184	1.090397	-1.165821
H	1.599545	2.705512	-0.523684
H	2.258481	-1.047129	-0.974358
H	2.494420	-1.747682	0.673083
H	1.615265	-2.668158	-0.589552

Conformer eee⁺

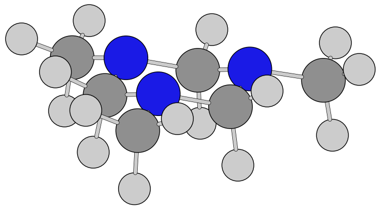

eee^{+}	Cartesian coordinates (Angstroms)		
Element	x	y	z
N	0.000000	1.396422	-0.263501
C	1.223554	0.706419	0.076516
N	1.209337	-0.698211	-0.263501
C	0.000000	-1.412838	0.076516
N	-1.209337	-0.698211	-0.263501
C	-1.223554	0.706419	0.076516
C	0.000000	2.830245	0.050899
C	2.451064	-1.415123	0.050899
C	-2.451064	-1.415123	0.050899
H	1.411644	0.815013	1.177617
H	2.065159	1.192320	-0.445955
H	-2.065159	1.192320	-0.445955
H	-1.411644	0.815013	1.177617
H	0.893029	3.288495	-0.396194
H	0.000000	3.018449	1.140742
H	-0.893029	3.288495	-0.396194
H	0.000000	-2.384641	-0.445955
H	0.000000	-1.630026	1.177617
H	2.401405	-2.417634	-0.396194
H	3.294435	-0.870861	-0.396194
H	2.614054	-1.509225	1.140742
H	-3.294435	-0.870861	-0.396194
H	-2.401405	-2.417634	-0.396194
H	-2.614054	-1.509225	1.140742

Conformer aee$^+$

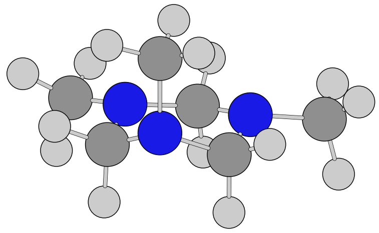

aee$^+$	Cartesian coordinates (Angstroms)		
Element	x	y	z
N	0.481560	1.501758	0.000000
C	-0.181338	1.006077	1.183540
N	-0.181338	-0.470968	1.216856
C	-0.510115	-1.146997	0.000000
N	-0.181338	-0.470968	-1.216856
C	-0.181338	1.006077	-1.183540
C	1.918842	1.170828	0.000000
C	-0.555460	-1.133670	2.463920
C	-0.555460	-1.133670	-2.463920
H	-1.226228	1.373763	1.211714
H	0.339246	1.353978	2.088674
H	0.339246	1.353978	-2.088674
H	-1.226228	1.373763	-1.211714
H	2.102033	0.077072	0.000000
H	2.385500	1.611514	0.891903
H	2.385500	1.611514	-0.891903
H	-0.058823	-2.159755	0.000000
H	-1.625251	-1.319547	0.000000
H	-0.437436	-2.221164	2.354219
H	0.110117	-0.777611	3.263387
H	-1.601663	-0.904675	2.739410
H	0.110117	-0.777611	-3.263387
H	-0.437436	-2.221164	-2.354219
H	-1.601663	-0.904675	-2.739410

Conformer tw-boat-eee$^+$

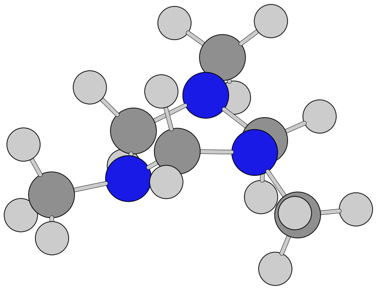

tw-boat-eee$^+$	Cartesian coordinates (Angstroms)		
Element	x	y	z
N	-0.624210	1.109229	-0.256503
C	-1.163014	0.097760	0.685581
N	-0.760192	-1.241393	0.376387
C	0.557093	-1.240426	-0.238327
N	1.352749	-0.127913	0.221810
C	0.826318	1.159901	-0.187962
C	-1.260589	2.431552	-0.168655
C	-1.752684	-2.067676	-0.320245
C	2.806374	-0.270916	0.072318
H	-2.256412	0.180628	0.744505
H	-0.703418	0.356102	1.672988
H	1.160189	1.929721	0.540241
H	1.212910	1.451285	-1.189884
H	-1.135184	2.876231	0.836228
H	-2.329195	2.330160	-0.399572
H	-0.792951	3.093400	-0.911721
H	1.074894	-2.196977	-0.070283
H	0.390174	-1.108163	-1.343710
H	-1.332623	-3.069459	-0.486695
H	-2.646188	-2.156091	0.312640
H	-2.031780	-1.617108	-1.293140
H	3.293539	0.616418	0.500257
H	3.135682	-1.158259	0.629087
H	3.100943	-0.368520	-0.989065

As part of the name of the conformer, tw-boat implies that the ring structure became a twisted boat structure upon optimization starting with a boat structure.
